# Awakening sleeper cells: a narrative review on bacterial magic spot synthetases as potential drug targets to overcome persistence

**DOI:** 10.1007/s00294-021-01221-z

**Published:** 2021-11-17

**Authors:** Vimal Venu Veetilvalappil, Jesil Mathew Aranjani, Fayaz Shaik Mahammad, Alex Joseph

**Affiliations:** 1 Department of Pharmaceutical Biotechnology, Manipal College of Pharmaceutical Sciences, Manipal Academy of Higher Education, Manipal, Udupi, Karnataka 576104 India; 2 Department of Biotechnology, Manipal Institute of Technology, Manipal Academy of Higher Education, Manipal, Udupi, Karnataka 576104 India; 3 Department of Pharmaceutical Chemistry, Manipal College of Pharmaceutical Sciences, Manipal Academy of Higher Education, Manipal, Udupi, Karnataka 576104 India

**Keywords:** Alarmone synthesis, Bacterial magic spots, Rel/SpoT homologs, Stringent response, Bacterial stress survival mechanism, Wake persister cells

## Abstract

Magic spot synthetases are emerging targets to overcome persistence caused by stringent response. The ‘stringent response’ is a bacterial stress survival mechanism, which results in the accumulation of alarmones (also called Magic spots) leading to the formation of dormant persister cells. These ‘sleeper cells’ evade antibiotic treatment and could result in relapse of infection. This review broadly investigates the phenomenon of stringent response and persistence, and specifically discusses the distribution, classification, and nomenclature of proteins such as Rel/SpoT homologs (RSH), responsible for alarmone synthesis. The authors further explain the relevance of RSH as potential drug targets to break the dormancy of persister cells commonly seen in biofilms. One of the significant factors that initiate alarmone synthesis is nutrient deficiency. In a starved condition, ribosome-associated RSH detects deacylated tRNA and initiates alarmone synthesis. Accumulation of alarmones has a considerable effect on bacterial physiology, virulence, biofilm formation, and persister cell formation. Preventing alarmone synthesis by inhibiting RSH responsible for alarmone synthesis will prevent or reduce persister cells’ formation. Magic spot synthetases are thus potential targets that could be explored to overcome persistence seen in biofilms.

## Introduction

"It is not the strongest of the species that survives; it is the one that is the most adaptable to change"—Charles Darwin

**Fig. 1:**
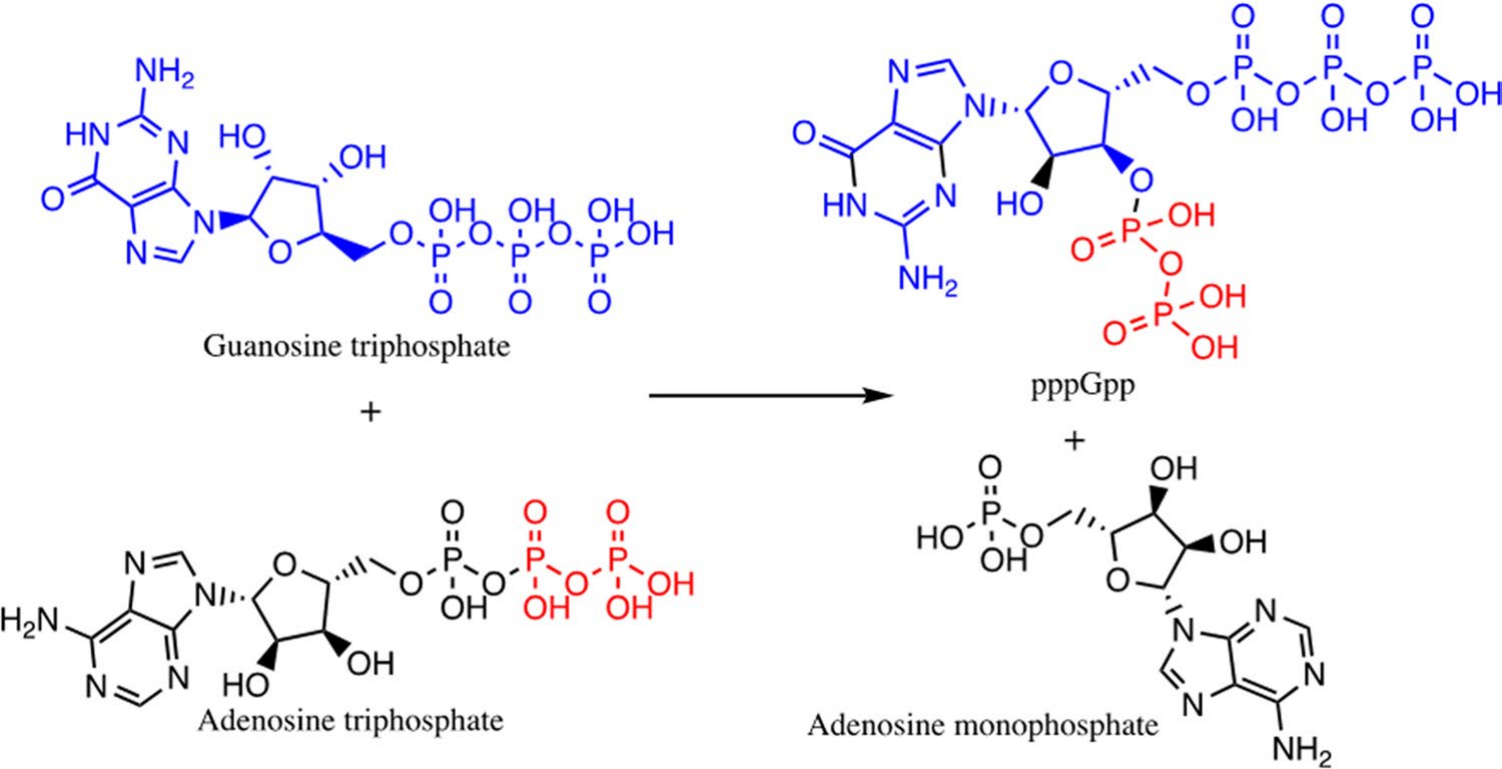
Synthesis of pppGpp from ATP and GTP. Synthesis occurs by the transfer of diphosphate from ATP to 3′-OH oxygen of GTP. Color indicates the transfer

All organisms must adapt to their environment to survive. Bacteria are endowed with several regulatory systems to adapt to an ever-changing environment around them. Some of these systems work at a transcriptional level, while others operate at a non-transcriptional level. Central long-term survival mechanisms exhibited by bacteria are stringent response (Irving et al. 2020), toxin–antitoxin system (Song and Wood 2020), SOS response (Podlesek and Bertok 2020), and oxidative stress response mechanism (Hong et al. 2019). Of these mechanisms, the stringent response is a pleiotropic stress response mechanism that helps bacteria survive during starvation (amino acids, fatty acids, or carbon sources). The stringent response results in the accumulation of bacterial second messengers called alarmones, and this accumulation results in drastic alteration in the bacterial physiology and metabolism needed for stress survival (Irving et al. 2020). The phenomenon of stringent response was discovered in *E. coli* by Stent and Brenner (1961) (3). Bacterial second messengers called alarmones or magic spots mediate stringent response. Cashel and Gallant (1969) first identified bacterial magic spots in 1969. They found two new spots in the thin layer chromatogram of acid-soluble metabolites of a stringent *E. coli *culture. The newly identified compounds were christened as Magic spot I and Magic spot II (MS I and MS II). Later these compounds were identified as guanosine 3′-diphosphate 5′-triphosphate (pppGpp) and guanosine 3′-diphosphate 5′-diphosphate (ppGpp) nucleotides. Recently, a third member of this group, guanosine 3′-diphosphate 5′-monophosphate (pGpp), has been confirmed (Yang et al. 2020a). All of them have been together referred to as (pp)pGpp. pppGpp, ppGpp, and pGpp are formed from GTP, GDP, and GMP, respectively, by transferring pyrophosphate moieties from ATP. The transfer of pyrophosphate groups occurs from ATP to the 3′-OH group of ribose of either GTP, GDP, or GMP. pGpp is also formed by hydrolysis of (p)ppGpp by the action of NahA enzyme in Bacillus species (Yang et al. 2020b). Figure 1 shows the formation of pppGpp from ATP and GTP. During the normal growth cycle of bacteria, (p)ppGpp is produced at a basal level and plays important role in maintaining GTP homeostasis, translation, transcription, and energy generation (Gaca et al. 2015a, b; Steinchen et al. 2020). Basal levels of alarmones are also required for expression of virulence factors and antibiotic tolerance (Abranches et al. 2009, 2006; Silva and Benitez 2006). During stress, over production of alarmones occur. This toxic accumulation of high levels (p)ppGpp during stress leads to a physiologically inactive state called persistence.

Bacterial recalcitrance to antibiotics is often attributed to genotypic resistance. However, phenotypic states such as persistence and tolerance can also contribute to antimicrobial tolerance, especially in chronic infections involving biofilms. These antibiotic-tolerant cells are called persisters. Persisters are a small subpopulation (0.001–1% of the total population) of antibiotic-tolerant but not resistant microbes (Balaban et al. 2019), usually protected deep within biofilms (Dewachter et al. 2019). Hobby et al. (1942) found that penicillin could kill only multiplying cells. Bigger (1944) confirmed persistence in 1944. In his study, he found that a subpopulation of *Staphylococcus aureus* had become tolerant to the antibiotic penicillin. The slow dividing nature of persister cells makes them tolerant to most antibiotics that target metabolically active cells (Girgis et al. 2012). Persistence can be triggered (type 1) or spontaneous (type 2). Type 1 persistence is initiated by a stress, like starvation or presence of an antibiotic or high cell density or presence of immune factors. Spontaneous persistence is seen in steady-state cultures and is less common than triggered persistence (Balaban et al. 2019). Triggered persistence is not a permanent phenomenon. Persister cells will revert to their usual selves whenever the persistence inducing stress (starvation, antimicrobials, oxygen depletion, thermal shock, pH variations, etc.) is removed. Persisters are considered as a subpopulation of tolerant bacteria and persistence is also known as heterotolerance (Balaban et al. 2019). Therefore, understanding the mechanism of persister cell formation and ways to prevent the formation or break the dormancy of already formed persister cell will be helpful in devising strategies for the prevention of chronic bacterial infections. The proteins responsible for alarmone synthesis may be potential targets to break the phenomenon of bacterial persistence. These proteins belong to a superclass of enzymes called RelA/SpoT homologs (RSH) or alarmone synthetases or magic spot synthetases.

This review investigates the phenomenon of stringent response and persistence; specifically discusses the distribution, classification, and nomenclature of proteins such as Rel/SpoT homologs (RSH); and explains the mechanisms by which alarmones can be targeted to eliminate persister cells in biofilms.

## Distribution of RSH

RSH are conserved in almost all bacteria and plant chloroplasts (Braeken et al. 2006, 2008; Masuda et al. 2008). Atkinson et al. analyzed 928 complete bacterial genome sequences and found that 92% of them carried genes encoding a long RSH. Most bacterial species have at least one RSH enzyme, except planctomycetes, verrucomicrobia, and chlamydiae (Atkinson et al. 2011; Hauryliuk and Atkinson 2017). A long Rel/SpoT homolog in plants was first reported in *Arabidopsis* *thaliana*. Van der Biezen et al. reported At-RSH1, 2, and 3, which showed significant homology to bacterial RSH in *A. thaliana* (Van Der Biezen and Jones 1998; Van Der Biezen et al. 2000). The presence of ppGpp in eukaryotes was first reported in *Drosophila melanogaster*, and scientists found a small alarmone hydrolase: Mesh1 (metazoan SpoT homolog 1) responsible for the hydrolysis of ppGpp. An analog of SpoT, namely human Mesh1 (hMesh1), has been identified in human cells, which is responsible for the activity of NADPH phosphatase that promotes ferroptosis (Ding et al. 2020). Both Mesh1 and hMesh1 are examples of small alarmone hydrolases. The presence of ppGpp in human cells was confirmed by Ito et al. (2020). However, the protein responsible for ppGpp synthesis in metazoans has yet to be confirmed. Bifunctional Rel is the most widely distributed alarmone synthetase in bacteria, while β and γ-proteobacteria encode Rel A and SpoT (Atkinson et al. 2011). It has been postulated that all RSH had a common ancestor, Rel protein, and gene duplication resulted in their diversification (Atkinson and Hauryliuk 2012). Table 1 gives information about RSH present in some common human pathogens.

**Table 1:** Alarmone synthetases present in common human pathogens

SI. no	Organism	Alarmone synthetase	Class
1	*Escherichia coli*	RelA	Monofunctional synthetase
		SpoT	Bifunctional RSH
2	*Staphylococcus aureus *(Siegmund et al. 2021)	Rel	Bifunctional RSH
		yjbm	Small alarmone synthetase
		ywac	Small alarmone synthetase
3	*Vibrio cholerae *(Dasgupta et al. 2014)	RelV	Small alarmone synthetase
		RelA	Monofunctional synthetase
		SpoT	Bifunctional RSH
4	*Bacillus subtilis *(Fung et al. 2020)	Rel	Bifunctional RSH
		yjbm	Small alarmone synthetase
		ywac	Small alarmone synthetase
5	*Enterococcus faecalis *(Gaca et al. 2015a, b)	Rel	Bifunctional RSH
		RelQ	Small alarmone synthetase
6	*Mycobacterium tuberculosis* (Bag et al. 2014)	Rel	Bifunctional RSH
7	*Pseudomonas aeruginosa *(Khakimova et al. 2013)	RelA	Monofunctional synthetase
		SpoT	Bifunctional RSH

## Nomenclature and classification of RSH

Wendrich et al. (2000) came up with a nomenclature system for the three long RSH present in bacteria. They named RelA as ppGpp synthetase I, SpoT as ppGpp synthetase II, and Rel as ppGpp synthetase III. According to their classification, RelA is monofunctional and synthesizes ppGpp during amino acid starvation. SpoT is responsible for the synthesis of ppGpp during carbon source starvation and is also responsible for the degradation of ppGpp (bifunctional). Bifunctional Rel can synthesize ppGpp during both amino acid and carbon starvation. It is also responsible for the degradation of ppGpp in a manganese-dependent manner.

**Fig. 2:**
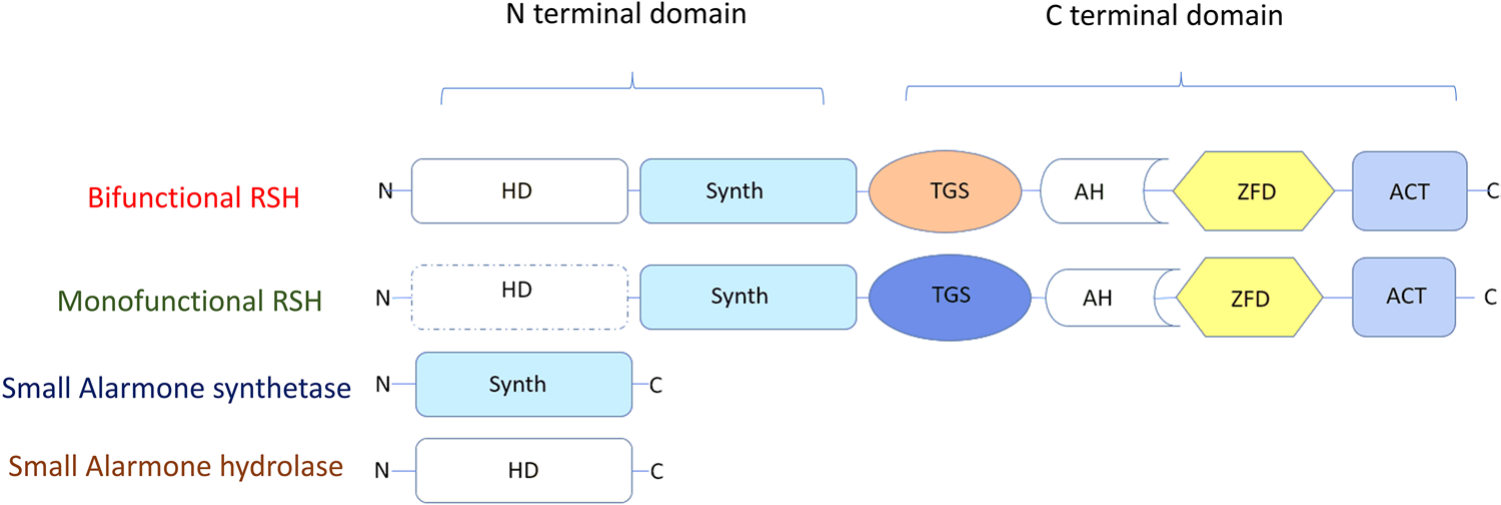
Domain organization of Rel/SpoT homologs. The hydrolase domain is not functional in monofunctional RSH. Solid shapes represent active domains. The dotted rectangle represents non-functional HD domain. Small alarmone synthetases and small alarmone hydrolases have synthetase and hydrolase domains, respectively. HD hydrolysis domain, Synth synthetase domain, *TGS* threonyl tRNA synthetase, GTPase, and SpoT, *AH* alpha helical, *ZFD* zinc finger domain, *ACT* aspartate kinase–chorismate mutase–TyrA domain.

**Fig. 3:**
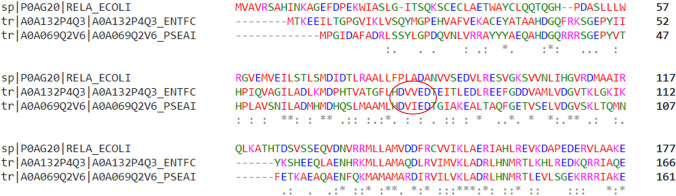
Result of multiple sequence alignment of HD domains of RelA of *Escherichia coli* (P0AG20), Rel of *Enterococcus faecium* (A0A132P4Q3), and, SpoT of *Pseudomonas aeruginosa* (A0A069Q2V6). It can be found that HDXXED motif (highlighted using a circle) is present in bifunctional Rel and SpoT. The absence of this motif in RelA is the reason behind lack of hydrolytic activity. (Asterisk) Multiple sequence alignment was done using Clustal Omega platform

Atkinson et al. classified RSH into 30 subgroups within 3 main groups: long RSH, small alarmone synthetases (SAS), and small alarmone hydrolases (SAH) (Atkinson et al. 2011). Long RSH have multidomain architecture, while SAS and SAH have a single domain organization (Fig. 2). Rel, RelA, and SpoT are examples of long RSH. The N-terminus end of these proteins carries a SYNTH/Synthetase domain and a hydrolase domain (HD) (Pausch et al. 2020). The C-terminus carries the zinc finger domain (ZFD) and aspartate kinase-chorismate mutase-TyrA (ACT)/RRM (ribosome recognition motif) subdomains. The terminals are connected by a middle region formed of the TGS domain and an alpha-helical (AH) region (Kushwaha et al. 2019a, b). The hydrolase domain is functional in Rel and SpoT; these enzymes show synthetic and hydrolytic activity (bifunctional) of ppGpp. RelA carries a pseudohydrolase domain and has only alarmone synthetic activity (monofunctional) (Irving et al. 2020). The absence of the HD*XX*ED motif in the HD domain is responsible for the lack of hydrolytic activity of monofunctional RelA (Fig. 3) (Aravind and Koonin 1998). SAS carry only the SYNTH domain and lack all other domains (Jimmy et al. 2019). Examples of SAS are RelQ/YjbM/SAS1/SasB, RelP/YwaC/SAS2/SasA present in firmicutes and RelV present in the genus Vibrio (Hauryliuk and Atkinson 2017; Irving and Corrigan 2018). The three-dimensional structure of SAS resembles the synthetase domain of long RSH (Kushwaha et al. 2019a, b). As the name indicates, SAH carry only the hydrolysis domain. Mesh1 and hMesh1 are examples of SAH. The most studied RSH are the monofunctional RelA of *E. coli.*

## Mechanism of alarmone synthesis (RelA–ribosome–deacylated tRNA complex formation)

The multidomain protein RelA exists in association with the ribosome. It identifies nutrient deficiency by detecting non-acylated tRNA at the ribosome’s A-site (Amino-acyl site) (Kudrin et al. 2018). In the absence of deacyl tRNA, it exists in a closed conformation, and hence there is no synthesis of (p)ppGpp. Usually, the catalytic N-terminal end of the ribosome extends out of the ribosome, while the regulatory C-terminal region is embedded inside the ribosome (Kushwaha et al. 2019a, b). Purified RelA has minimal alarmone synthetic activity in vitro, which suggests the importance of its binding to deacylated tRNA and ribosome (Jenvert and Schiavone 2005). The unbound RelA exists in a closed conformation, devoid of synthetic activity. This closed conformation is caused by the interaction (oligomerization) of the RelA CTD with the synthetase domain in NTD (Mechold et al. 2002). The amino acid residues Cys-612, Asp-637, and Cys-638 are essential in this downregulation (Gropp et al. 2001). The formation of disulfide bonds between cysteine residues plays a significant role in this negative regulation of synthetic activity. Yang and Ishiguro (2001) attributed the negative regulatory activity of the C-terminal domain to homodimer formation. They proved that two C-terminal regions: regions from amino acids 455–538 and amino acids 550–682, were responsible for dimerization. In addition to dimer formation, region 550–682 is the main ribosomal binding region of RelA. The CTD of RelA binds to L11 protein and nucleotides 1051–1108 of 23S rRNA of 50S subunit (Agirrezabala et al. 2013). Gropp et al. (2001) proposed that this dimer is enzymatically inactive. Therefore, during the nonstarved condition, RelA exists as an oligomer (specifically a dimer) and is inactive.

Whenever a deacylated tRNA enters the 30S A-site of the ribosome, conformational changes occur, which results in alarmone synthesis (Loveland et al. 2016). The TGS subdomain and α-helical region wraps around deacylated tRNA and convert it into a unique conformation called A/R conformation, accommodated at the A-site of the ribosome (Brown et al. 2016; Loveland et al. 2016; Arenz et al. 2016). In this open conformation, the RelA–ribosome–deacylated tRNA complex starts the synthesis of alarmones. According to this popular model, alarmone synthesis requires uncharged tRNA at the A-site of the ribosome bound to mRNA (Haseltine and Block 1973). Wendrich et al. showed that the synthesis of ppGpp was also dependent on the ribosomal protein L11 (Wendrich et al. 2002). The interaction between the L11 protein and the distorted tRNA at the A-site is required to activate the synthetic activity of RelA. They found that ribosomes deficient in the L11 protein, a critical component of the 50S ribosome, were inactive in the synthesis of ppGpp. The presence of an optimal concentration of Mg^2+^ ions is also needed for alarmone synthesis. It was also found that at high concentrations, Mg^2+^ ions would inhibit alarmone synthesis in bifunctional Rel. Surprisingly this inhibitory effect was not seen in monofunctional RelA. Sajish et al. (2007) proposed that the substitution of the R*X*KD motif in the synthetase domain of Rel by the E*X*DD motif in RelA is the reason for this difference. After synthesis of alarmone, RelA gets released from the ribosome, and it hops to the next stalled ribosome to continue alarmone synthesis. Hence, this model is known as the “hopping model” (Wendrich et al. 2002).

English et al. (2011) proposed an “extended hopping model,” in which RelA retained its catalytic activity even after dissociation from the ribosome. Winther et al. (2018) came up with an alternative model of complex formation. According to their model, the RelA–deacylated tRNA complex is formed in the cytoplasm, away from the ribosome, and this integrates with the ribosome to create the final complex.

## Synthetase domain and ppGpp synthesis

**Fig. 4:**
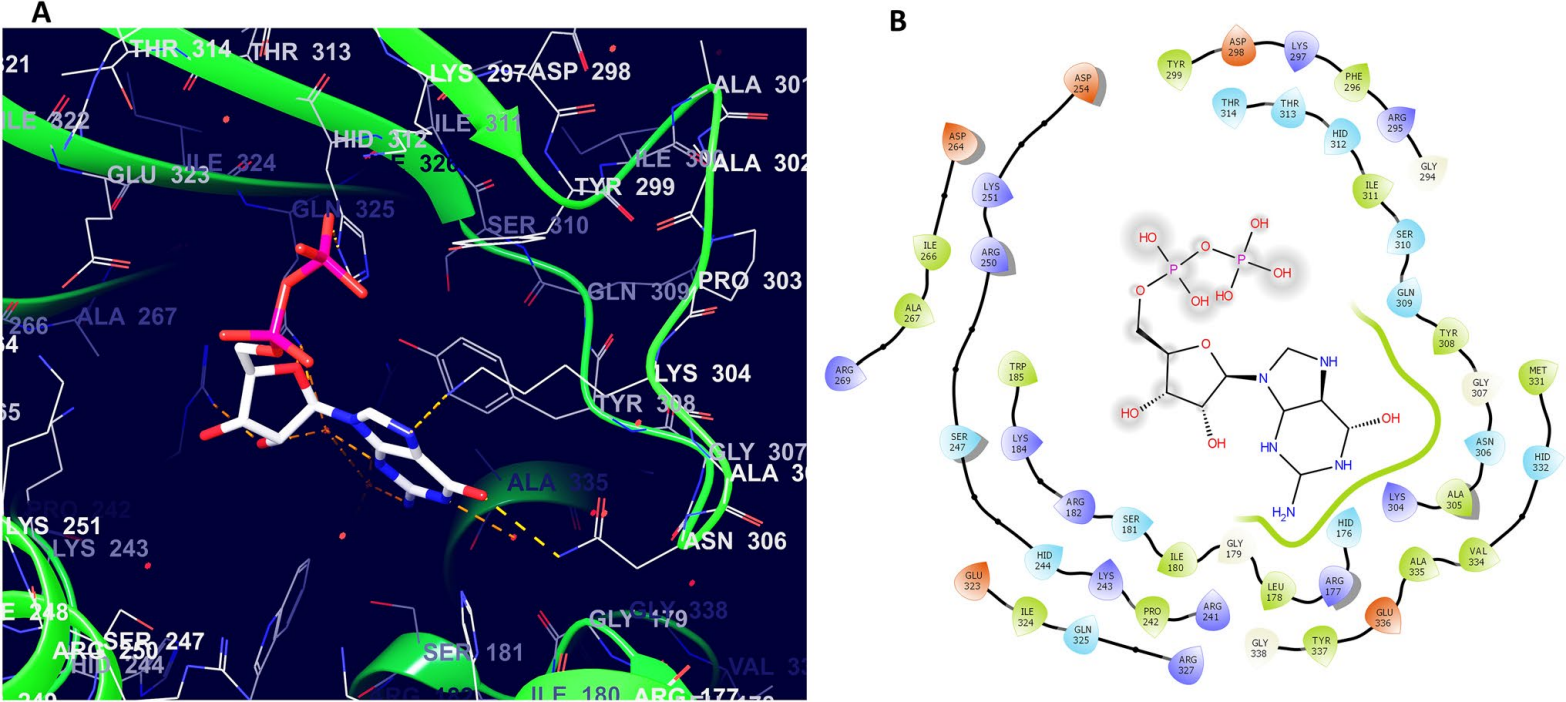
**A** The 3D representation of the ligand GDP within the binding pocket of Rel of Streptococcus equisimilis. **B** The different amino acid residues surrounding the ligand GDP in the synthetase region of Rel of Streptococcus equisimilis. (Asterisk) Structures were taken from Protein data bank and processed using Schrodinger™ software

We have seen the classification of RSH in the preceding section. Despite the differences in domain organization, the active site responsible for alarmone synthesis is commonly seen in all RSH (Patil et al. 2020). ATP, GTP/GDP, and the catalytic Mg^2+^ bind to different sites of the synthetase domain to carry out alarmone synthesis. Synthesis and hydrolysis of alarmones are performed by two distinct subdomains present in NTD of Rel protein, namely the synthetase domain and hydrolase domain. The hydrolase domain is inactive in RelA due to the absence of the HD*XX*ED motif (Aravind and Koonin 1998; Irving and Corrigan 2018). The Synthetase/SYNTH subdomain is present in the NTD of RelA of *E. coli*, and it extends from amino acids 181–372 (Brown et al. 2016). Different motifs present in the synthetase domain play a pivotal role in alarmone synthesis. Sajish et al. (2009) studied the importance of conserved motifs E*X*DD and R*X*KD present in ppGpp synthetases. Bifunctional RSH has an R*X*KD motif in the synthetase domain, while monofunctional RSH has an E*X*DD motif. They found that the specificity of the GTP/GDP substrate (EXDD containing RelA preferred GDP over GTP), response to Mg^2+^ ions, regulation of catalytic activity by C-terminal domain depended on this motif difference. Patil et al. (2020) proved the catalytic role of 2′-OH of GTP in alarmone synthesis by providing a pivotal hydrogen bond. The binding sites for substrates are present in the synthetase domain. *Streptococcus equisimilis* Rel (Rel_Seq_), the substrate-binding site, comprises amino acid residues Arg241, Lys243, Arg269, Lys297, Tyr299, Lys304, Asn306, Tyr308, His312, Glu323, Arg327, Ala335, and Glu336 (Kushwaha et al. 2019a, b). Figure 4 shows the binding pocket for GDP present in synthetase region of Rel of *S. equisimilis*.

## Effects of alarmones on bacterial physiology, virulence, and biofilm formation

During the stress response, alarmones accumulate inside the bacterial cell. This results in many transcriptional level changes. These changes result in the repression of growth and cell division and activate genes responsible for the biosynthesis of amino acids, acquisition of nutrients, and virulence (Chang et al. 2002; Durfee et al. 2008; Geiger et al. 2010; Chatnaparat et al. 2015; Frank et al. 2014; Vogt et al. 2011). The molecular basis of the stress response varies in Gram-negative and Gram-positive microbes. Different types of Gram-negative pathogens also show diversity in their regulatory mechanisms. Gammaproteobacteria, such as *E. coli*, use the sigma subunit σ^S^/σ^38^/RpoS as the primary regulator of stress response, while alphaproteobacteria depend on the PhyR sigma factor (Hengge 2014). Through direct binding to RNA polymerase (RNAP) and interaction with the transcriptional regulator DksA, alarmones cause downregulation of several growth-promoting genes in *E. coli* (Ross et al. 2016). Indirect transcriptional control occurs by repression of the housekeeping sigma factor (σ^70^) (Bernardo et al. 2006; Szalewska-Palasz et al. 2007). Direct and indirect mechanisms in *E. coli* cause downregulation of stable RNA biosynthesis, inhibition of DNA replication, downregulation of cell wall, and phospholipid biosynthesis resulting in growth arrest (Braeken et al. 2006). In Gram-positive organisms, alarmones do not interact directly with RNAP. In them, ppGpp causes changes by altering the nucleotide pool. Increased alarmone synthesis is associated with a decreased level of intracellular GTP in firmicutes such as *S. aureus *and* Bacillus subtilis. *(p)ppGpp inhibits Gmk, HprT and XPRT essential for GTP synthesis which results in a decrease in GTP concentration (Kriel et al. 2012; Anderson et al. 2020). Reduced GTP levels results in reduced rRNA expression and growth arrest (Anderson et al. 2021).

Bacteria show several traits that help them invade the host and establish an infection. These are known as virulence factors. Alarmones play a vital role in the regulation of virulence factors (Dalebroux et al. 2010). In *Pseudomonas aeruginosa*, alarmones are essential in the full expression of the *rhl* and *las* quorum sensing systems, which play a vital role in the expression of virulence (Schafhauser et al. 2014). Erickson et al. found that the *P. aeruginosa* *relA*-mutant strain showed attenuated virulence in a *D. melanogaster* infection model (Erickson et al. 2004). *Yersinia pestis relA/spot* mutants exhibited decreased amounts of plasmid-encoded virulence proteins associated with the type 3 signaling system (T3SS) (Sun et al. 2009). Aberg et al. (2006) found that ppGpp-deficient uropathogenic *E. coli* (UPEC) failed to produce type 1 fimbriae needed for initial attachment to bladder cells. They also found that such strains had reduced ability to form biofilms. RSH mutant strains of *Enterococcus faecalis*, *E. coli*, *Streptococcus mutans*, and *Vibrio cholerae* had attenuated virulence and exhibited defective biofilm formation (Balzer and McLean 2011; Lemos et al. 2004; He et al. 2012; Moyed and Bertrand 1983)*.*

## Alternative determinants of bacterial persistence 

After the discovery of persistence, many studies were conducted to understand the molecular basis of persistence. A well-studied mechanism for persister formation is the toxin–antitoxin (TA) system (Page and Peti 2016). TA systems play a significant role in the formation of both biofilms and persister cells (Wang and Wood 2011). A toxin is activated following a stress, whose expression inhibits physiological processes. In the HipBA system, stress degrades the antitoxin HipB, which causes expression of toxin HipA. This results in suppression of translation factor EF-Tu leading to growth arrest (Schumacher et al. 2009). Kaspy et al. (2013) found that expression of HipA caused phosphorylation of enzyme GltX, which in turn resulted in an accumulation of uncharged tRNA inside cell. This accumulation triggered stringent response. Norton et al. found that the pasTI TA system increased persister formation in extraintestinal pathogenic *E. coli* (ExPEC), which are significant causative microbes of sepsis, urinary tract infections, and meningitis (Norton and Mulvey 2012). Other TA systems which may have an impact on persister cells’ formation are MqsR/Mqs (Kim and Wood 2010), higB, MazF, yafQ and yaeB in *E. coli* (Shah et al. 2006) and MazE, RelE (Lemos et al. 2005). Mutations increase frequency of persister cells’ formation. Moyed et al. (1983) found that high persistence mutations (*hip* mutations such as *hipA7*) increased the frequency of persister cell formation by 10,000 times in *E. coli*). Wolfson et al identified another high persistence gene* HipQ* in *E. coli. *They found that HipQ mutant strain exhibited high persistence in the presence of ampicillin and norfloxacin (Wolfson et al. 1990). Korch et al. (2003) demonstrated that RSH knockouts eliminated persistence in *hipA7*-mutant strains, which meant that hipA7 mutation induced persistence by over production of alarmones by RSH. Another mechanism that resulted in persistence during the stationary phase was the RpoS-mediated general stress mechanism. *RpoS *is a gene that encodes a unique sigma factor called RpoS. The accumulation of RpoS inside cells causes the cells to enter the stationary phase (Battesti et al. 2011). Studies have shown that ppGpp is one of the factor controlling the expression of RpoS during stationary phase (Spira and Ospino 2020; Hirsch and Elliott 2002). It is inferred that during starvation there is over production of ppGpp, which causes overexpression of *RpoS* and subsequent development of persistence. According to Conlon et al. (2016) depletion of ATP could produce persister cells in *S. aureus*. They found that deleting TA modules did not have any effect on persister cell formation in *S. aureus*. This means that toxin–antitoxin systems do not play role in persister formation in Gram-positive *S. aureus*, unlike Gram-negative *E. coli*. Variations in ATP levels might also play a role in persister cells’ generation. As a conclusion, it can be inferred that a multitude of factors contribute to the onset of persistence. More studies are required to conclude which is the most critical mechanism controlling stringent response and how these systems are inter-related.

## Magic spot synthetases as a potential drug target to overcome persistence

To date, two different approaches have been explored to block stringent response mechanisms in bacteria. The first approach involved the discovery of molecules that degrade already formed alarmones. Fuente-Nez et al. synthesized an antibiofilm cationic peptide 1018 (VRLIVAVRIWRR-NH) in 2014, which blocked the stringent response. This peptide caused the degradation of alarmones formed within cells. Peptide 1018 prevented biofilm formation and eradicated biofilms formed in Gram-negative and Gram-positive pathogens such as *P. aeruginosa*,* Escherichia coli*,* Acinetobacter baumannii*,* Klebsiella pneumoniae*, methicillin-resistant *S. aureus (MRSA)*, *Salmonella typhimurium* and *Burkholderia cenocepacia *(Battesti et al. 2011; Trastoy et al. 2018; de la Fuente-Núñez et al. 2014). In 2015, D-amino acid peptides were made, which were analogs of peptide 1018, which potentiated antibiofilm activities of conventional antibiotics up to almost 64 times (Trastoy et al 2018). These peptides were able to prevent *P. aeruginosa* infections (de la Fuente-Núñez et al. 2014, 2015). A major feature of these peptides is that they acted against biofilms produced by both Gram-positive and Gram-negative microbes, but had a high MIC (minimum inhibitory concentration) against planktonic forms (Fuente-Núñez and Hancock 2015). Peptide 1018 had a MIC of 64 µg/ml against *P. aeruginosa *and 128 µg/ml against *A. baumannii*. Cost of production and stability of peptides in the presence of host proteases are other concerns in developing these peptides as therapeutic agents. Adding to these concerns, a later study proved that peptide 1018 lacked specificity towards ppGpp (Andresen et al. 2016).

The second approach involved designing molecules that inhibited the proteins (RSH) responsible for alarmone synthesis. Most of them were structural modifications of ppGpp. Wexselblatt et al. (2010), synthesized a group of ppGpp analogs known as ‘Wexselblatt’s Bisphosphonates,’ which competitively inhibited RelA activity in vitro. The compound 2-deoxyguanosine-3′-5′-di (methylene bisphosphonate) was found to inhibit RSH in Gram-positive and Gram-negative microbes. Although the introduction of methylene bridges between phosphate groups considerably improved stability, the molecule required 1 mM concentration for 50% inhibition of *E. coli* RelA in vitro. The hydrophilic nature of molecule rendered it ineffective against live microbes (Beljantseva et al. 2017). Wexselblatt et al. (2012), synthesized a novel antibacterial agent targeting stringent response and named it relacin. It was synthesized by substituting the 3′ and 5′ end of ppGpp with glycyl-glycine dipeptides. They studied the inhibitory potential of this compound on GTP pyrophosphokinases from *E. coli* and *B. subtilis*. In *E. coli*, although it showed inhibitory activity in vitro, there was no in vivo activity, which was due to the inability of relacin to cross the bacterial cell wall. But it was found that relacin inhibited both in vitro and in vivo ppGpp synthesis in *B. subtilis. *In *B. subtilis*, relacin inhibited the formation of biofilm pellicles, as well as the formation of spores (Wexselblatt et al. 2012). When compared to bisphosphonate, relacin had lower hydrophilicity and showed in vivo action against *B. subtilis* which was a considerable improvement. Still its potency was not substantial (IC_50_ = 200 µM) and had no biological activity against Gram-negative microbes (Chau et al. 2021). The quest for more potent molecules started by keeping either relacin or ppGpp as the base molecule.

Wexselblatt et al. (2013), synthesized eight structural analogs of relacin and studied their effects on purified GTP pyrophospho kinases from *E. coli* and *D. radiodurans*. The compound that they named “2d”, which had two glutamyl-glutamic acid moieties at the 3′ and 5′ end of deoxyribose, showed better in vitro activity than relacin (Wexselblatt et al. 2013)

Syal et al. (2017a, b) prepared four synthetic analogs of ppGpp by acetylation and benzoylation reactions. They found that the compound, which was acetylated and benzoylated, inhibited the formation of biofilms and disrupted the formed biofilms in *Mycobacterium smegmatis.* Syal et al. also found that vitamin C at high concentration could inhibit in vivo (p)ppGpp formation in *M. smegmatis.* Vitamin C in high concentration could stall long-term survival and biofilm formation in *M. smegmatis* (Syal et al. 2017a, b). Pandit et al found that low concentrations of vitamin C (upto 30 µM) could also hinder biofilm formation by decreasing the synthesis of extra polymeric matrix (Pandit et al. 2017).

In 2019, Dutta et al. virtually screened the GlaxoSmithKline (GSK) compound library that contained 2 million compounds against the Rel protein of *Mycobacterium tuberculosis.* The compound that showed the most promising inhibitory activity was named “X9”. It was found to increase the susceptibility of *M. tuberculosis* to Isonicotinic acid hydrazide (INH) treatment. This showed the potential of Rel inhibitors as potential re-sensitizers of conventional antibiotics (Dutta and Klinkenberg 2019). In Hall et al. (2020) screened the ZINC database using the RelA protein structure from the Protein data bank (PDB ID:5IQR). They came up with two molecules: S3-G1A and S3-G1B, having better docking scores than relacin. Both compounds also showed better in vitro activity. These compounds showed no activity against planktonic cells. From the study, it was also found that the hit compounds reduced the formation of biofilm matrix. An Alamar blue viability assay showed that ampicillin killed more *E. coli* cells when combined with lead molecules.

## Conclusion

Conventional antibiotics kill planktonic cells, but their inability to terminate the dormant persister cells often results in the relapse of infection more severely. To break this phenotypic resistance, new molecules are needed that will eliminate persister cells and inhibit biofilm formation. Magic spot synthetases are such proteins whose inhibition will result both in biofilm inhibition and prevention of persister cell survival. The discovery of such molecules will help in the re-sensitization of antibiotics which are losing their relevance in treating biofilm-related chronic infections. Combining Rel inhibitors with antibiotics will provide a new platform in treating relapsing infections caused by bacterial pathogens.

## Data Availability

Data sharing is not applicable to this article as no datasets were generated or analyzed.
